# Dual-mechanism of phytochemicals in cancer therapy: synergistic anti-angiogenic potency in cancer and proangiogenic impact against chemotherapy-induced cytotoxicity in vital organs

**DOI:** 10.3389/fphar.2026.1783390

**Published:** 2026-03-23

**Authors:** Deiaa E. Elsayed Abouzed, Nervana M. K. Bayoumy, Hanan Hagar, Safwat M. Rabea, Hamada Hashem, Hazim O. Khalifa

**Affiliations:** 1 Department of Pharmacology and Toxicology, Faculty of Pharmacy, Sohag University, Sohag, Egypt; 2 Department of Physiology, College of Medicine, King Saud University, Riyadh, Saudi Arabia; 3 Department of Biology, University of Arkansas at Little Rock, Little Rock, AR, United States; 4 Department of Pharmacology, College of Pharmacy, Zagazig University, Zagazig, Egypt; 5 Medicinal Chemistry Department, Faculty of Pharmacy, Minia University, Minia, Egypt; 6 Pharmaceutical Chemistry Department, Faculty of Pharmacy, Sohag University, Sohag, Egypt; 7 Department of Veterinary Medicine, College of Agriculture and Veterinary Medicine, United Arab Emirates University, Al Ain, United Arab Emirates

**Keywords:** angiogenesis, antiangiogenetic therapy, chemotherapy toxicity, cytoprotection, phytochemicals

## Abstract

Angiogenesis is essential for tumor growth and metastasis, yet physiological angiogenesis is equally critical for tissue repair and endothelial homeostasis. This creates a therapeutic dilemma: suppressing tumor neovascularization without impairing vascular integrity in healthy organs, particularly under cytotoxic chemotherapy. This review summarizes key pro- and anti-angiogenic pathways that regulate tumor vascularization, highlights limitations of current anti-angiogenic therapies (including resistance and delivery barriers), and synthesizes evidence that selected phytochemicals exert *context-dependent angiogenesis modulation*. Specifically, many compounds inhibit pathological tumor angiogenesis (via VEGF/HIF-1α/NF-κB/MMP-related signaling) while supporting endothelial defense and microvascular recovery in non-cancerous tissues through antioxidant, anti-inflammatory, and anti-apoptotic pathways (e.g., Nrf2/HO-1). Finally, we discuss translational requirements—bioavailability, standardization, dosing windows, and safety—to inform rational adjunct development and future biomarker-driven clinical trials.

## 1 Introduction

Cancer is the primary cause of morbidity and death globally, and its progression is intriguingly associated with hemodynamics (Santucci et al., 2020; Torre et al., 2016). After a certain size, tumors need a steady flow of oxygen and nutrients to develop and spread. Tumor angiogenesis, a process that creates new blood vessels to meet this need, is controlled by a careful balancing act between pro- and anti-angiogenic processes (Yang et al., 2018; Nguyen-Kim et al., 2015). Cancer progression depends on the acquisition of a functional blood supply, enabling tumors to overcome diffusion limits for oxygen and nutrients and to facilitate metastatic dissemination. Tumor angiogenesis is regulated by a dynamic balance between pro-angiogenic mediators (e.g., VEGF family ligands, FGFs, angiopoietins) and endogenous anti-angiogenic signals. In malignant disease, this balance shifts toward a sustained pro-angiogenic state (‘angiogenic switch’), resulting in structurally and functionally abnormal vasculature that contributes to hypoxia, therapeutic resistance, and metastatic potential (Hoff and Machado, 2012; Saman et al., 2020). From basic blood vessel flexibility in reaction to vasodilators and vasoactive gases to endothelial cell migration and the development of new lumens, angiogenesis encompasses a wide range of physiological processes that have been documented (Papetti and Herman, 2002; Dudley and Griffioen, 2023). This information is mediated by well-known proangiogenic mediators such as Vascular Endothelial Growth Factor (VEGF) and Basic Fibroblast Growth Factor (BFGF) (Karamysheva, 2008; Kuwano et al., 2001). Proangiogenic factors promote tumor-associated angiogenesis, while anti-angiogenic mechanisms, comprising natural inhibitors such as angiostatin and endostatin, act as natural barriers against uncontrolled angiogenesis (Hong and Park, 2022; Anakha et al., 2023). It turned out that tumors act as a biological barrier against -capable of increasing angiogenesis, and hence, their blood supply. This is believed to be controlled by the p53 gene and happens early in the course of cancer (Duffy et al., 2014). From this knowledge came the hypothesis that blocking lymphatic vessels could inhibit tumor growth and cause tumor regression due to a lack of nutrients and oxygen (Ioannidou et al., 2021). The development of this hypothesis led to research and experiments on antiangiogenic agents. The therapeutic potential of targeting angiogenesis in cancer has contributed to the development of various antiangiogenic therapies, which show promising results as adjuvants to combination therapy with chemotherapy and radiation therapy, which requires further understanding.

### 1.1 Novelty and scope of this review

Unlike prior reviews that primarily catalogue anti-angiogenic phytochemicals in oncology, this article synthesizes evidence supporting a *dual, context-dependent angiogenesis-modulating paradigm*: (i) suppression of pathological tumor angiogenesis and vascular support of metastasis, and (ii) preservation or restoration of physiological endothelial integrity and microvascular repair in chemotherapy-injured vital organs. We further integrate mechanistic targets with organ-specific protection data and discuss translational constraints (bioavailability, dosing windows, standardization, and safety), thereby providing a framework to guide rational adjunct use and the design of biomarker-informed clinical trials.

## 2 Methodology

To create this comprehensive review on the proangiogenic and anti-angiogenic pathways, plus the role of antiangiogenic agents in cancer management, a systematic literature search was carried out across multiple online databases, including PubMed, Elsevier, Google Scholar, Egyptian Knowledge Bank (EKB), Embase, and Web of Science. The strategy of the search included a combination of relevant keywords and subject headings linked to angiogenesis, pro-angiogenic factors, anti-angiogenic mechanisms, antiangiogenic phytochemicals, cytoprotective phytochemicals, and cancer therapy. Inclusion criteria were established to select original research papers and clinical studies published in peer-reviewed journals over the past decade in the English language only. A critical study and synthesis of the selected literature was carried out to provide readers with a comprehensive understanding of the current state of knowledge and recent advancements in this subject.

## 3 Results

### 3.1 Angiogenesis and cancer

For angiogenesis, cancer progression, and metastasis, major considerations exist. Since metastasis is the advancement of cancer from its original place to other parts of the body, it is the primary cause of death for cancer patients (Dillekås et al., 2019). Angiogenesis is now generally acknowledged to be essential for tumor development and advancement to a clinically identified condition. This was demonstrated by a 1989 study that showed that lymph nodes provided a local route to tumor cells (Williams et al., 1989). Through specific proliferative factors, tumor cells can induce a vascular response that provides an important mechanism for proliferation. Research using animal models has demonstrated that anti-angiogenics could effectively prevent vascular metastasis by inhibiting tumor formation (Teleanu et al., 2019). This has now led to the idea that angiogenesis is a feasible target for the treatment plus prevention of cancer metastasis, thereby improving patient survival.

The relationship between the number of cancers and their progression can be partially linked to the growth ability of tumor blood vessels. The angiogenesis process is regulated by stimulators and inhibitors normally found in the body. In most angiogenic cases, stimulators surpass the inhibitors, which contribute to the formation of new blood vessels (Karamysheva, 2008). New cancer treatments have been developed because of greater knowledge of the impact of angiogenesis on tumors. It is currently being investigated and developed for use as an angiogenesis inhibitor to prevent the formation of additional blood vessels.

### 3.2 Mechanism of angiogenesis

**FIGURE 1:**
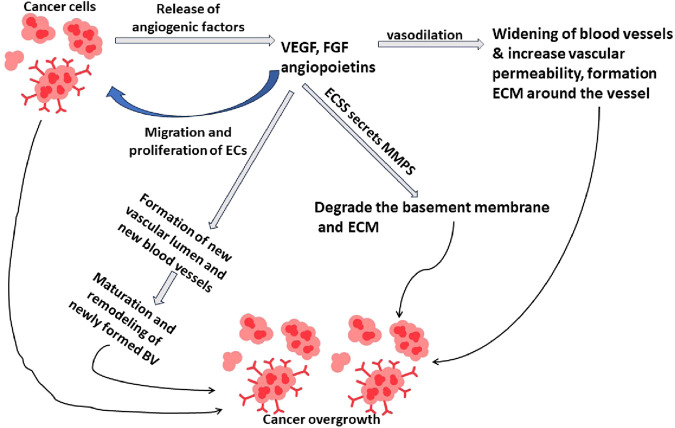
Mechanism of angiogenesis in cancer overgrowth.

Pathways of angiogenesis involve multiple steps and are mediated by various signaling molecules, growth factors, cytokines, and cellular interactions. As summarized in Figure 1, tumor-driven angiogenesis proceeds through a series of sequential steps, including the release of pro-angiogenic mediators (e.g., VEGF/FGF/angiopoietins), endothelial activation, matrix remodeling, sprouting and migration, lumen formation, and subsequent vessel maturation and stabilization (Karamysheva, 2008). In tumor cells, Angiogenic signaling mediators, like VEGF, FGF, and angiopoietins, are released, initiate and enlarge blood vessels, which will supply them with oxygen and nutrients to grow and metastasize. Moreover, angiogenic signals lead to vasodilation and increased vascular permeability. This allows for the leakage of plasma proteins, including fibrinogen, to form a provisional extracellular matrix (ECM) around the vessel. Furthermore, Endothelial cells (ECs) release proteolytic enzymes, such as matrix metalloproteinases (MMPs), that degrade the basement membrane and ECM surrounding the existing blood vessels. This facilitates the movement of ECs from the parent channel into the tissues around it. Additionally, ECs are prompted by angiogenic factors to move and proliferate (divide) in the direction of the angiogenic signal source. Migrating ECs start to organize into tubular structures, forming a new vascular lumen. Cell-to-cell contacts and the recruitment of auxiliary cells, like smooth muscle or pericytes, to stabilize the newly created vessels are involved in this process. Following this, the acquisition of pericytes, the formation of an additional basement membrane, and the establishment of functional linkages with the pre-existing vascular network are all steps in the maturation and remodeling process of newly produced blood vessels. This makes it possible for the surrounding tissues to receive oxygen and nutrients efficiently (Lugano et al., 2020).

#### 3.2.1 Role of vascular endothelial growth factors (VEGFs)

Vascular Endothelial Growth Factors (VEGFs) play a clinical function in the initiation, growth, and advancement of various types of cancer (Yang and Cao, 2022). VEGFs are a family of signaling proteins that encourage the synthesis of new blood vessels (Zhou et al., 2021). Formation of new blood vessels is essential for the development and spread of solid tumors because it provides the fast-dividing cancer cells with the oxygen and nutrients they need. VEGFs play specific roles in cancer, including tumor angiogenesis. As potent angiogenic factors, VEGFs stimulate the proliferation of endothelial cells, their migration and survival, and the formation of new blood vessels within the tumor. Because it gives the tumor oxygen and nutrition and encourages elasticity to get rid of metabolic wastes, this mechanism is crucial for tumor growth (Ghalehbandi et al., 2023). Angiogenesis also has an important function in the metastatic process. Cancer cells can enter the bloodstream through the new blood vessels and travel to other parts of the body, which leads to metastatic lesions (Yang and Cao, 2022). Furthermore, VEGF can promote tumor cell survival and resistance to treatments. They can protect tumor cells from apoptosis (programmed cell death) and make them more resistant to chemotherapy and radiation therapy (Harmey and Bouchier‐Hayes, 2002; Sharma et al., 2021). Also, VEGFs may contribute to an immunosuppression of the tumor microenvironment by inhibiting the growth and function of dendritic cells that are critical for initiating an effective immune response (Ribatti, 2022). In addition to blood vessel formation, VEGF can also enhance the synthesis of new blood vessels (lymph angiogenesis), allowing tumor cells to spread into lymph nodes and contribute to further metastasis (Kong et al., 2020).

Due to their potential role in tumor growth and progression, in cancer treatment, VEGF and its receptors are now crucial therapeutic targets. Several anti-angiogenic agents have been developed that target VEGF signaling for tumor angiogenic inhibition (Elebiyo et al., 2022; Masłowska et al., 2021; Ribatti et al., 2021). These medications are used in conjunction with other cancer treatments like chemotherapy or treatments for cancers like breast, lung, kidney, and colorectal cancer.

#### 3.2.2 Role of transforming growth factor-beta 1 (TGF-β1)

Transforming growth factor-beta 1 (TGF-β1) plays a complex and context-dependent role in the regulation of angiogenesis, particularly in cancer progression (Waldner and Neurath, 2012). Its effects on angiogenesis are highly variable and depend on multiple factors, including the stage of tumor development, the composition of the tumor microenvironment, and the presence and relative abundance of other signaling molecules. As a result, TGF-β1 can exert both pro-angiogenic and anti-angiogenic functions.

Under pro-angiogenic conditions, TGF-β1 promotes angiogenesis by stimulating tumor cells and stromal cells within the tumor microenvironment to increase the expression of key angiogenic mediators, including vascular endothelial growth factor (VEGF), fibroblast growth factor (FGF), and platelet-derived growth factor (PDGF) (Ferrara and Angiogenesis, 2019). In addition, TGF-β1 can directly activate endothelial cells, enhancing their migration and proliferation, which are critical early steps in new blood vessel formation. TGF-β1 also contributes to vascular maturation by recruiting and activating perivascular supporting cells, such as pericytes and smooth muscle cells, which guide vessel organization and stabilize newly formed vasculature.

Conversely, TGF-β1 can exert anti-angiogenic effects under certain conditions, particularly at elevated concentrations (Trinh et al., 2009). In such contexts, TGF-β1 suppresses endothelial cell proliferation and migration, thereby inhibiting angiogenic sprouting. Moreover, it can induce apoptosis in endothelial cells, leading to regression of existing blood vessels. TGF-β1 further antagonizes angiogenesis by upregulating endogenous angiogenesis inhibitors, including endoglin and thrombospondin-1 (TSP-1), which counteract the pro-angiogenic signaling of other growth factors. Collectively, these opposing actions highlight the dual and tightly regulated role of TGF-β1 in angiogenesis and underscore its significance as a context-dependent modulator of tumor vascularization.

### 3.3 Antiangiogenetic mechanism

On the other hand, antiangiogenic mechanisms include endogenous inhibitors such as angiostatin, endostatin, and thrombospondin 1, which cancel the effects of proangiogenic factors and inhibit steps of the angiogenic process. These inhibitors can reduce EC proliferation, migration, and tube formation, respectively, inducing endothelial cell apoptosis, ultimately preventing tumor angiogenesis, as previously published in 2019 by Li. Other colleagues reported that 90% of patients with colon cancer could die because of metastatic progression of the disease, and that the possibility of metastatic disease increases with the presence of high tumor vascularity (Li et al., 2017). This indicates that inhibition of tumor angiogenesis would mitigate the rate of mortality for these patients, as well as the overall mortality of cancers with similar progression rates.

#### 3.3.1 Attenuation of the VEGF signaling pathway

The attenuation of the VEGF signaling pathway has a primary function in the treatment of tumors by targeting the angiogenesis process (Waldner and Neurath, 2012). VEGF is a key signaling protein that initiates angiogenesis (Ferrara and Angiogenesis, 2019). It attaches specific receptors on the surface of ECs, mainly VEGFR-1 (Flt-1) and VEGFR-2 (KDR/Flk-1), initiating a sequence of signaling events inside the cell. When VEGF binds to its receptors, it stimulates multiple signaling pathways such as PI3K/Akt and MAPK, which encourage endothelial cell growth, movement, and the synthesis of new blood vessels (Trinh et al., 2009). By inhibiting VEGF, we can put the brakes in this process, making it harder for cancer cells to invade new tissues and establish secondary tumors. But there’s another benefit too. VEGF pathway inhibition can also partially normalize abnormal tumor vasculature, which may improve perfusion and enhance delivery of cytotoxic agents, thereby augmenting combination therapy efficacy. VEGF inhibitors can help “normalize” these vessels, allowing treatments to be delivered more successfully to the tumor cells. So not only do these inhibitors slow the spread, but they also boost the impact of other therapies (Wu et al., 2018).

#### 3.3.2 Disruption of tumor blood supply

Antiangiogenic agents have become a promising addition to cancer treatment, working by targeting different parts of the blood vessel formation process. These agents generally fall into two main groups: first, there are monoclonal antibodies and small molecule inhibitors that focus on blocking proangiogenic factors like VEGF and its receptors (Wahl et al., 2011). The second group either mimics the body’s natural anti-angiogenic substances or interferes with other pathways involved in angiogenesis (Guan et al., 2016). Both approaches aim to cut off the blood supply that tumors rely on, helping to slow down their growth.

### 3.4 Anti-angiogenic drugs and their clinical applications in cancer treatment

Several anti-angiogenic drugs, including bevacizumab, a monoclonal antibody targeting vascular endothelial growth factor A (VEGF-A), as well as small-molecule tyrosine kinase inhibitors such as sunitinib and sorafenib, have been developed for the treatment of various solid tumors, including colorectal, lung, renal, and hepatocellular carcinomas. These agents have demonstrated improved clinical outcomes, particularly when used in combination with conventional chemotherapy or other targeted therapies. In this section, selected anti-angiogenic drugs and their clinical applications in cancer treatment are discussed.

#### 3.4.1 Bevacizumab (Avastin)

Bevacizumab is a monoclonal antibody that acts on VEGF, a key angiogenic factor. It is approved for the treatment of certain types of cancers, including colorectal cancer (Krämer and Lipp, 2007), non-small cell lung cancer (Wang et al., 2020), breast cancer (Cameron and Bell, 2008), glioblastoma, ovarian cancer (Loizzi et al., 2017), renal cell carcinoma (Yang et al., 2003), etc. Chemotherapy and other targeted treatments are frequently used in conjunction with bevacizumab.

#### 3.4.2 Sunitinib (Sutent)

Sunitinib is a tyrosine kinase inhibitor with a small molecular weight that targets several angiogenesis-related tissues (Hao and Sadek, 2016), including VEGF receptors (Roskoski, 2007), platelet growth factor receptors (PDGFRs) (Christensen, 2007), and stem cell factor receptor (KIT) (Brossa et al., 2015), renal cell carcinoma (Tamaskar et al., 2008), gastrointestinal stromal tumors (GISTs) (Seandel et al., 2006).

#### 3.4.3 Sorafenib (Nexavar)

Sorafenib is a novel tyrosine kinase inhibitor with a small molecular weight that targets VEGF receptors (Wang et al., 2016), PDGFRs (Wilhelm et al., 2008), and RAF kinase (Wilhelm et al., 2008). It is approved for the management of advanced renal cell carcinoma (Tamaskar et al., 2008), hepatocellular carcinoma (Liu et al., 2012), and thyroid cancer (Corrado et al., 2017).

#### 3.4.4 Pazopanib (Votrient)

A multitargeted tyrosine kinase inhibitor, pazopanib, inhibits c-KIT, PDGFRs, and VEGF receptors. It is authorized to treat smooth muscle sarcoma and advanced renal cell carcinoma (Schutz et al., 2011; Jones et al., 2022).

#### 3.4.5 Ramucirumab (Cyramza)

A monoclonal antibody called ramucirumab targets the VEGF receptor 2 (VEGFR-2) (Fornaro et al., 2022). Along with other medications, it is authorized to treat colorectal cancer (Ren et al., 2024), non-small cell lung cancer, and advanced stomach or esophageal adenocarcinoma (Hochster et al., 2024).

#### 3.4.6 Aflibercept (Zaltrap)

A recombinant fusion protein that binds to placental growth factor (PlGF), VEGF-A, and VEGF-B and functions as a decoy receptor. It can be used in conjunction with chemotherapy to treat metastatic colorectal cancer (Zhao et al., 2022; Hong et al., 2020; Wang et al., 2021).

#### 3.4.7 Conbercept (Lumitin)

A VEGF-Trap fusion protein that connects to and inhibits VEGF-A, VEGF-B, and PlGF. It is accredited for the management of age-associated macular degeneration and diabetic macular edema in some nations (Liu et al., 2021; Liu et al., 2020).

It is essential to observe that these anti-angiogenic substances are often utilized in conjunction with other treatment options, such as chemotherapy, targeted therapies, or immunotherapies, as part of a multi-modality approach to treating most cancers. The choice of anti-angiogenic agent and treatment regimen depends on factors that include the type and stage of cancer, as well as patient-specific factors.

### 3.5 Phytochemicals as antiangiogenic and cytoprotective for vital organs in cancer treatment

**FIGURE 2:**
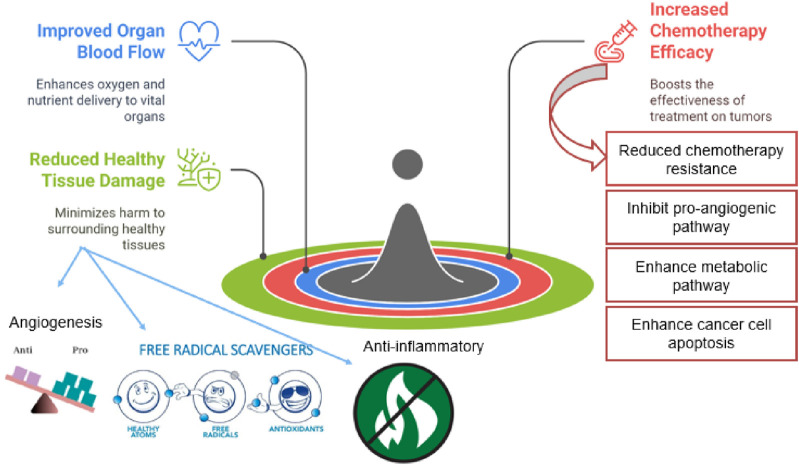
Anticancer effects of phytochemicals.

A growing body of evidence indicates that numerous plant-derived phytochemicals possess significant anti-angiogenic properties and have been extensively investigated for their potential role in cancer therapy (Fu et al., 2015) (Figure 2). To improve clarity and comparability, each phytochemical subsection is organized into three parts: (i) tumor antiangiogenic/anticancer actions, (ii) evidence for organ cytoprotection and microvascular repair in chemotherapy-induced injury models, and (iii) interaction with chemotherapy, including reported synergy, sensitization, or formulation approaches that may influence efficacy and safety.

**FIGURE 3:**
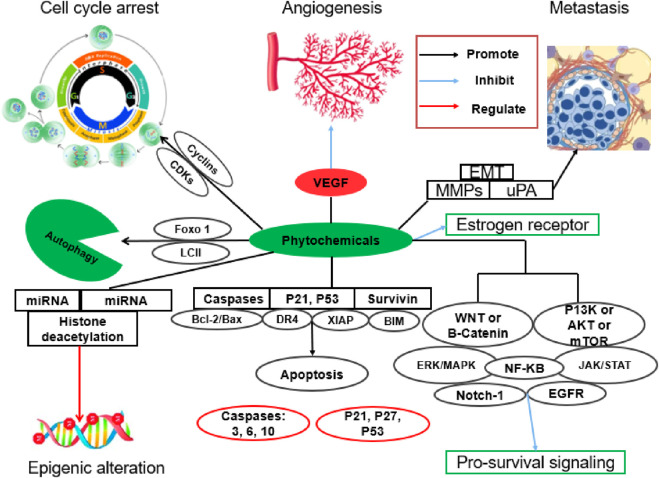
Phytochemical potential in chemotherapy modified after (Usman et al., 2022).

Notably, many of these compounds exhibit dual functionality by inhibiting pathological angiogenesis within tumors while simultaneously supporting or preserving physiological angiogenesis in vital organs. This dual action may contribute to cytoprotective effects, helping to mitigate chemotherapy-induced vascular and tissue damage in non-cancerous organs (Huang et al., 2017) (Figure 3). Table 1 summarizes representative phytochemicals and their angiogenesis-related anticancer activities.

**TABLE 1:** Selected phytochemicals targeting angiogenesis in cancer therapy.

Phytochemical	Plant source	Mechanism of anti-angiogenesis	Cancer type studied	References
Curcumin	Turmeric (*Curcuma longa*)	Reduced VEGF expression, downregulates NF-kB	Breast, colorectal carcinoma	Zhang et al. (2021), Huang et al. (2017), Banerjee et al. (2016)
Resveratrol	Grapes (*Vitis vinifera*)	Inhibits HIF-1α and VEGF, induces apoptosis in endothelial cells	Prostate and breast cancer	Delmas et al. (2011), Fu et al. (2021)
Epigallocatechin-3-gallate (EGCG)	Green tea (*Camellia sinensis*)	Inhibits VEGF, inhibits MMP-2 and MMP-9	Lung, prostate cancer	Yamakawa et al. (2004), Demeule et al. (2000)
Genistein	Soybeans (*Glycine max*)	Inhibits VEGF and EGFR signaling	Breast, prostate cancer	Varinska et al. (2015), Guo et al. (2007), Hassanshahi et al. (2019)
Quercetin	Apples, onions	Inhibits VEGF, reduces MMP activity	Lung, prostate cancer	Zhang et al. (2021), Zhang et al. (2022)
Berberine	Goldenseal (*Hydrastis canadensis*)	Suppresses HIF-1α, inhibits VEGF	Breast, ovarian cancer	Fu et al. (2013), Yin et al. (2021)
Sulforaphane	Broccoli (*Brassica oleracea*)	Inhibits HDAC, downregulates VEGF	Prostate, breast cancer	Russo et al. (2018), Pledgie-Tracy et al. (2007)
Withaferin A	*Withania somnifera*	Downregulates VEGF	Pancreas, ovary cancer	Lee and Choi (2016), Atteeq (2022), Sharma et al. (2017)

#### 3.5.1 Curcumin

##### 3.5.1.1 Tumor antiangiogenic/Anticancer actions

The active compound in turmeric (Curcuma longa) exhibits antiangiogenic activity by inhibiting endothelial proliferation, migration, and tube formation, and by suppressing key proangiogenic mediators, particularly VEGF and inflammatory transcriptional programs such as NF-κB (Mohammadi et al., 2022; Van Der Vlies et al., 2019; Gururaj et al., 2002; Zhang et al., 2021; Fu et al., 2015; Huang et al., 2017). Pimentel-Gutiérrez et al. (2016) reported that curcumin potentiates the chemotherapy effect against acute lymphoblastic leukemia through the mitigation of the NF-KB pathway (Pimentel-Gutiérrez et al., 2016). In addition, Rudnik et al. (2020) reported that co-loaded curcumin and methotrexate nanocapsules enhanced cytotoxicity against non-small-cell lung cancer cells (Rudnik et al., 2020).

##### 3.5.1.2 Organ cytoprotection during chemotherapy-related injury

**FIGURE 4:**
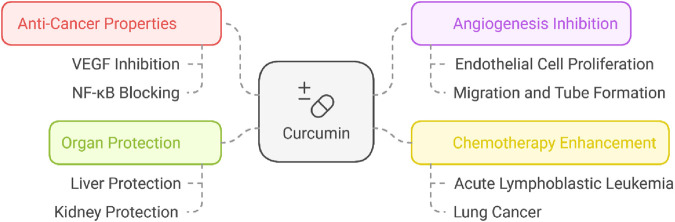
Curcumin’s dual role in cancer treatment and protection of vital organs.

Beyond tumor-directed effects, curcumin demonstrates organ-protective activity in chemotherapy injury models, with reported hepatoprotective effects against cisplatin-associated injury and protection against methotrexate/cyclophosphamide-associated liver toxicity, as well as renoprotection in methotrexate-related toxicity models (Commandeur and Vermeulen, 1996; Liu et al., 2018). While it is busy cutting off the blood supply to tumors and fighting inflammation, curcumin can shield healthy tissues from chemo’s toxic side effects, making it a double win in cancer treatment. Wang et al. (2014) documented that curcumin exhibited hepatoprotection against cisplatin-induced hepatotoxicity. Furthermore, Banerjee et al. (2016) reported that curcumin exhibited hepatoprotective effects against methotrexate and cyclophosphamide-induced liver toxicity (Banerjee et al., 2016). In addition, curcumin protects the kidneys against methotrexate toxicity (Morsy et al., 2013) (Figure 4).

##### 3.5.1.3 Combination with chemotherapy

Collectively, these data support curcumin as a candidate adjunct whose antiangiogenic tumor mechanism may coexist with organ protection, although translation requires careful attention to dosing windows, formulation strategies, and interaction monitoring.

#### 3.5.2 Resveratrol

##### 3.5.2.1 Tumor antiangiogenic/Anticancer actions

Resveratrol, a polyphenol abundant in grapes (*Vitis vinifera*), has been studied for anti-inflammatory, antioxidant, and anticancer properties. In tumor-relevant settings, resveratrol exhibits antiangiogenic activity through inhibition of HIF-1α and VEGF signaling, particularly under hypoxic conditions characteristic of many tumors (Wong and Fiscus, 2015; Yun et al., 2021; Aggarwal et al., 2004). On top of that, resveratrol triggers apoptosis (cell death) in endothelial cells, which helps block the formation of new blood vessels that tumors need to grow (Delmas et al., 2011; Fu et al., 2021). Santandreu et al. (2011) documented that resveratrol potentiates the oxidative damage effect of chemotherapy in colorectal cancer management (Santandreu et al., 2011).

##### 3.5.2.2 Organ cytoprotection during chemotherapy-related injury

**FIGURE 5:**
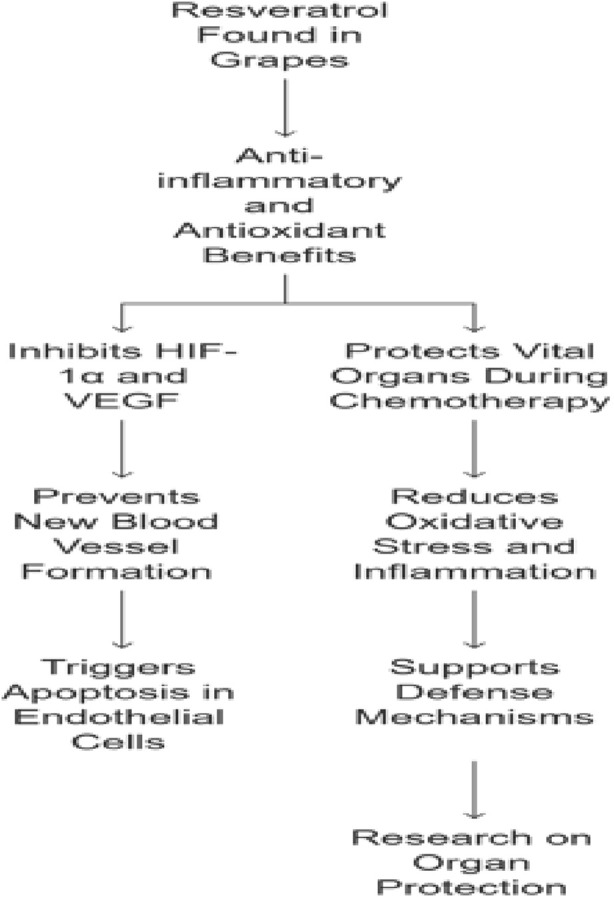
Resveratrol dual role in cancer treatment and protection of vital organs.

Beyond tumor-directed activity, resveratrol has been associated with protecting vital organs during chemotherapy exposure (Patra et al., 2021; Xiao et al., 2019). Chemotherapy, while essential for targeting and destroying cancer cells, can take a heavy toll on healthy organs like the heart, liver, and kidneys (Koivusalo and Hietanen, 2004; Osborne et al., 1989). Resveratrol helps to reduce the damage caused by chemotherapy (Mortezaee et al., 2020). By neutralizing harmful oxidative stress and inflammation, resveratrol supports the body’s defense mechanisms, potentially easing some of the negative side effects of chemotherapy (Shi et al., 2018). It is like having a double layer of protection when it is needed most. Kaffashielahi, (2014) documented that resveratrol protects the liver against the hepatotoxic effect of cisplatin (Kaffashielahi, 2014). Meghji et al. (2019) reported that resveratrol prevents acute kidney failure induced by chemotherapy via attenuating oxidative damage in the kidneys (Meghji et al., 2019). Multiple studies have also described cardioprotective effects against chemotherapy-induced cardiotoxicity (Ranjan et al., 2021; Abdelgawad et al., 2019; Wang et al., 2009) (Figure 5).

##### 3.5.2.3 Combination with chemotherapy

Resveratrol has been investigated as an adjunct that may modulate chemotherapy response, including potentiation of oxidative damage mechanisms in colorectal cancer models. Collectively, these findings position resveratrol as a candidate dual-action adjunct; nonetheless, careful study design is required to confirm that normal-tissue protection does not inadvertently reduce tumor sensitivity, particularly across different doses and treatment schedules.

#### 3.5.3 Epigallocatechin-3-gallate (EGCG)

##### 3.5.3.1 Tumor antiangiogenic/anticancer actions

EGCG, the most abundant catechin in green tea (*Camellia sinensis*), has demonstrated antiangiogenic actions relevant to cancer progression. EGCG can downregulate VEGF signaling, thereby limiting a key pathway tumors use to promote neovascular growth (Shankar et al., 2012; Jung et al., 2001). Gu et al. (2013) disclosed that EGCG attenuated breast cancer via the antiangiogenic pathway and reduced the activation of HIF-1α and NFκB, and VEGF expression (Gu et al., 2013). Furthermore, the enzymes matrix metalloproteinases (MMP-2 and MMP-9) that degrade extracellular matrix and promote the migration of endothelial cells, which line new blood arteries, are inhibited by EGCG (Yamakawa et al., 2004; Demeule et al., 2000). By disrupting these processes, EGCG helps prevent the formation of new blood vessels in tumors, making it a potent agent against cancer progression in various types of cancers (Fang et al., 2015). Interestingly, EGCG potentiates the cytotoxic effect of chemotherapeutic agents in cancer treatment (Wei et al., 2020; Hu et al., 2015; Sehgal et al., 2023). Chen et al. (2020) documented that a combination of Cisplatin/EGCG exhibits a synergistic action in the management of lung cancer (Chen et al., 2020).

##### 3.5.3.2 Organ cytoprotection during chemotherapy-related injury

**FIGURE 6:**
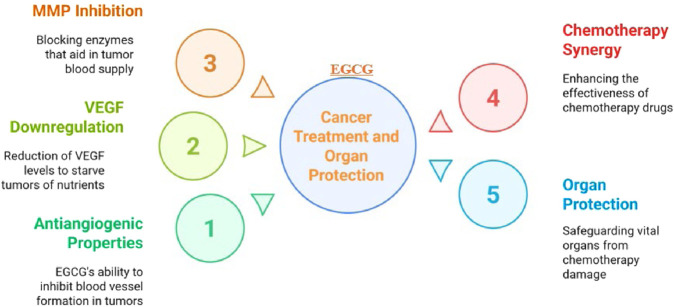
Epigallocatechin-3-gallate (EGCG) dual role in tumor treatment and protection of vital organs.

In non-cancerous tissues exposed to chemotherapy stress, EGCG has been associated with endothelial protection and organ resilience, consistent with antioxidant and anti-inflammatory activity. This is crucial because, while blocking blood vessel growth in tumors helps starve cancer cells, supporting healthy blood flow in vital organs like the heart, kidneys, and liver is equally important, especially during the stress of chemotherapy. Chemotherapy, while potentially effective at killing cancer cells, often injures healthy cells too, leading to organ toxicity and harmful side effects. EGCG helps counteract this by encouraging blood vessel formation in non-cancerous tissues, improving oxygen and nutrient supply to these organs, which aids in repair and recovery. Tucker et al. (2014) reported that EGCG diminished angiogenesis in melanoma without affecting the angiogenesis and VEGF pathway in skeletal muscles and heart (Tucker et al., 2014). Moreover, EGCG’s antioxidant and anti-inflammatory properties further protect these organs from the cytotoxic effects of chemotherapy. Pradhan et al. (2023) documented that EGCG protects the liver against methotrexate induced hepatotoxicity (Pradhan et al., 2023). Furthermore, Fatima et al. (2021) reported that EGCG protects the liver against cisplatin-induced liver injury (Fatima et al., 2021). Additionally, Pan et al. (2015) reported that EGCG reduces oxidative/nitrative damage, inflammation, and NF-κB expression, while stimulating the Nrf2/HO-1 signaling pathway, thereby ameliorating cisplatin-induced renal injury (Pan et al., 2015; Sahin et al., 2010). Interestingly, Ibrahim et al. (2019) reported that EGCG protects the heart from the toxic effects of cisplatin (Ibrahim et al., 2019). Collectively, these findings support a dual profile in which EGCG attenuates tumor-associated angiogenic signaling while mitigating chemotherapy-associated organ injury via antioxidant and anti-inflammatory mechanisms (Figure 6).

##### 3.5.3.3 Combination with chemotherapy

EGCG has been studied in combination with several chemotherapeutic agents and has been reported to potentiate cytotoxic activity in selected cancer models. Overall, the combination literature supports continued evaluation of EGCG as an adjunct, with attention to dose, timing, and formulation factors that may determine whether tumor-directed activity and organ protection are both achieved.

#### 3.5.4 Genistein

##### 3.5.4.1 Tumor antiangiogenic/Anticancer actions

Genistein, an isoflavone abundant in soybeans, has been studied for antiangiogenic and anticancer actions in hormone-associated malignancies. Mechanistically, genistein can interfere with VEGF-related signaling and has also been linked to EGFR pathway modulation, providing two relevant nodes for limiting tumor proliferation and vascular support (Spagnuolo et al., 2015; Varinska et al., 2015; Guo et al., 2007; Banerjee et al., 2008). By interfering with these signaling systems, genistein slows down the creation of new blood vessels and the uncontrolled maturation of cancer cells, which makes it particularly promising in managing hormone-dependent cancers like breast and prostate cancer (Varinska et al., 2015; Guo et al., 2007). Previous works documented that genistein enhanced sensitivity to cisplatin in breast cancer, ovarian cancer, colon cancer, and pancreatic cancer (Solomon et al., 2008; Hu et al., 2014; Banerjee et al., 2007). Mohamed et al. (2004) reported that diffuse large cell lymphoma is made more sensitive to CHOP (cyclophosphamide, doxorubicin, vincristine, and prednisone) chemotherapy by genistein (Mohammad et al., 2003).

##### 3.5.4.2 Organ cytoprotection during chemotherapy-related injury

**FIGURE 7:**
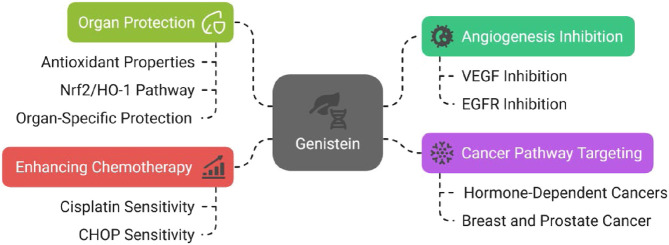
Genistein’s dual role in cancer treatment and protection of vital organs.

Genistein has also been studied as an organ-protective adjunct during cytotoxic therapy. Its antioxidant capacity and anti-inflammatory effects have been proposed as key contributors to tissue protection. Genistein acts as a powerful antioxidant (Mazumder and Hongsprabhas, 2016), which helps protect tissues from oxidative stress and inflammation (Goh et al., 2022). Sung et al. (2008) reported that genistein protected the kidney against cisplatin-induced renal injury (Sung et al., 2008). Another findings imply that genistein can shield bone marrow sinusoids from methotrexate therapy, which is linked, at least in part, to its direct action of increasing nitric oxide generation in SECs and its indirect effect of encouraging the expression of VEGF in osteoblasts (Hassanshahi et al., 2019). Chen et al. (2019) reported that genistein protected cardiac muscle against doxorubicin-induced cardiac toxicity via mediation of the Nrf2/HO-1 pathway (Chen et al., 2019). Mansour et al. (2017) documented that genistein protected the liver against cyclophosphamide-induced liver toxicity via modulation of oxidative damage and pro-inflammatory mediators (Mansour et al., 2017) (Figure 7).

##### 3.5.4.3 Combination with chemotherapy

The combined evidence indicates that genistein may function as both a chemosensitizer and an organ-protective adjunct in selected settings. Future work should clarify the dose-schedule relationship that best preserves tumor sensitivity while maximizing organ protection, particularly in multi-drug regimens.

#### 3.5.5 Quercetin

##### 3.5.5.1 Tumor antiangiogenic/Anticancer actions

Quercetin, a dietary flavonoid abundant in foods such as apples and onions, has been reported to inhibit angiogenesis-relevant signaling by lowering VEGF levels and suppressing MMP activity (Li et al., 2015). Since both VEGF and MMP play key roles in supporting tumor growth, quercetin’s impact in reducing their influence makes it a promising candidate for cancer therapy (Uttarawichien et al., 2021). Li et al. (2018) documented that quercetin potentiates the doxorubicin effect against breast cancer and protects the vital organs against the cytotoxic effects of doxorubicin (Li et al., 2018). Lee et al., (2015) disclosed that quercetin enhanced the chemotherapeutic effect of gemcitabine against lung cancer via attenuating the heat shock protein 70 expression (Lee et al., 2015). Chuang-Xin et al. (2012) documented that quercetin enhanced the effect of 5-fluorouracil in inhibiting the growth of colorectal cancer via attenuation of the NF-KB signaling pathway (Chuang-Xin et al., 2012).

##### 3.5.5.2 Organ cytoprotection during chemotherapy-related injury

**FIGURE 8:**
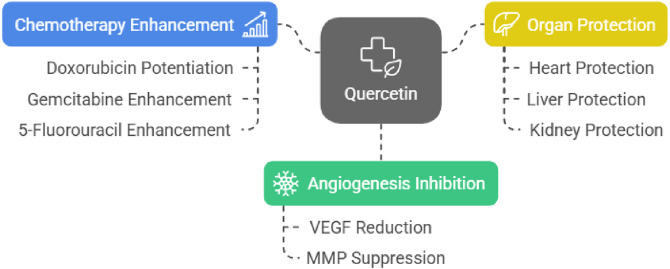
Quercetin’s dual role in cancer treatment and protection of vital organs.

Quercetin has additionally been reported to reduce chemotherapy-related organ injury, consistent with antioxidant and anti-inflammatory properties that mitigate cytotoxic stress. This protective capability enhances its appeal as a complementary treatment in cancer care, helping patients tolerate aggressive therapies more effectively. Zhang et al. (2022) reported that quercetin protects the heart against doxorubicin–cyclophosphamide during the management of triple-negative breast cancer (Zhang et al., 2022). Quercetin protects the liver against methotrexate-induced hepatotoxicity (Aydın, 2011; Kocahan et al., 2017). Quercetin protects the kidneys against methotrexate-induced renal toxicity (Kocahan et al., 2017; Erboga et al., 2015) (Figure 8).

##### 3.5.5.3 Combination with chemotherapy

Taken together, quercetin is supported by preclinical evidence as a candidate adjunct that may combine tumor-related antiangiogenic actions with organ protection. Translational evaluation should incorporate careful monitoring of exposure and interactions, particularly in multi-agent chemotherapy regimens.

#### 3.5.6 Berberine

##### 3.5.6.1 Tumor antiangiogenic/anticancer actions

Berberine, an alkaloid derived from *Hydrastis canadensis* and other botanical sources, has been studied for antiangiogenic activity in cancer models. Reported mechanisms include downregulation of HIF-1α and inhibition of VEGF expression, both of which are central drivers of tumor vascular support and metastatic potential (Lin et al., 2004; Pan et al., 2017; Fu et al., 2013; Yin et al., 2021). Studies have highlighted berberine’s potential in breast and ovarian cancers, positioning it as a promising adjunct to conventional therapies for these malignancies (Kaboli et al., 2014; Patil et al., 2010).

##### 3.5.6.2 Organ cytoprotection during chemotherapy-related injury

**FIGURE 9:**
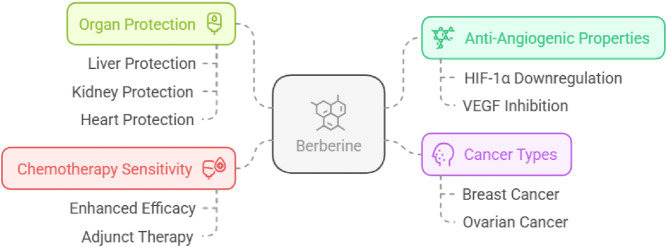
Berberine’s dual role in cancer treatment and protection of vital organs.

Berberine has also been associated with attenuation of chemotherapy-related organ toxicity and oxidative injury, with reported protection of the liver, kidney, and heart in experimental settings. Prior works documented the berberine sensitivity of chemotherapeutic agents in cancer management (Devarajan et al., 2021; Bharti et al., 2018; Anis et al., 1999). Chen et al. (2016) documented that berberine protected the liver and kidneys against the cytotoxic effect of doxorubicin (Chen et al., 2016). Hao et al. (2015) reported that berberine protected the heart against cardiotoxicity induced by doxorubicin by inhibiting doxorubicin metabolism (Hao et al., 2015). Berberine protects the liver against doxorubicin-induced hepatotoxicity (Zhao et al., 2012; Sun et al., 2020). Berberine showed protective benefits against free radical damage to cardiac tissue caused by DOX, possibly by reducing mitochondrial dysfunction and inhibiting intracellular Ca^2+^ increase (Xiong et al., 2018) (Figure 9).

##### 3.5.6.3 Combination with chemotherapy

Prior work has also suggested that berberine can influence chemotherapy responsiveness in tumor models. Because berberine may interact with drug metabolism pathways, future translational work should explicitly evaluate pharmacokinetic interactions and define schedules that preserve antitumor efficacy while maximizing organ protection.

#### 3.5.7 Sulforaphane

##### 3.5.7.1 Tumor antiangiogenic/Anticancer actions

Sulforaphane, a bioactive compound found in cruciferous vegetables such as broccoli (*Brassica oleracea*), has been reported to exert antiangiogenic activity by inhibiting HDAC and suppressing HIF-1α and VEGF signaling, pathways closely linked to tumor angiogenesis and adaptive survival (Kim et al., 2015; Russo et al., 2018; Pledgie-Tracy et al., 2007). Prior works documented that sulforaphane potentiates the chemotherapeutic power of cisplatin or doxorubicin (Negrette-Guzman, 2019). Rong et al. (2020) documented that sulforaphane enhanced the potential of doxorubicin in attenuating the growth of breast cancer by breaking the accumulation of myeloid-derived suppressor cells (Rong et al., 2020). By destroying cancer stem cells, sulforaphane increases the effectiveness of taxanes against triple-negative breast cancer (Burnett et al., 2017).

##### 3.5.7.2 Organ cytoprotection during chemotherapy-related injury

**FIGURE 10:**
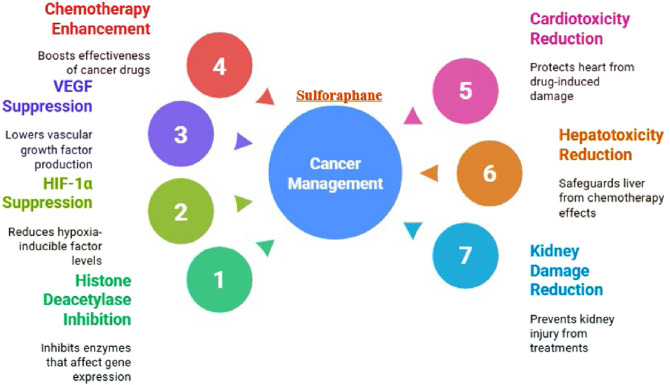
Sulforaphane dual role in cancer treatment and protection of vital organs.

Sulforaphane has also been reported to reduce chemotherapy-induced organ toxicity in models of cardiac, hepatic, and renal injury (Guerrero-Beltrán et al., 2012; Calcabrini et al., 2020; Cascajosa-Lira et al., 2024). Sulforaphane attenuates doxorubicin-induced cardiotoxicity (Singh et al., 2015). Sulforaphane attenuates cisplatin-induced hepatotoxicity (Gaona-Gaona et al., 2011). Sulforaphane decreased cisplatin-induced kidney damage by controlling the activation of many cell death and pro-inflammatory pathways (p53, JNK, p38-α, TNF-α, and NF-κB) and inhibiting crucial pro-survival signaling pathways (ERK and p38-β) (Guerrero-Beltrán et al., 2012) (Figure 10).

##### 3.5.7.3 Combination with chemotherapy

Sulforaphane has been reported to potentiate the anticancer activity of cisplatin and doxorubicin in selected models. Rong et al. (2020) documented enhanced doxorubicin efficacy against breast cancer through disruption of myeloid-derived suppressor cell accumulation. Together, these findings support sulforaphane as a candidate adjunct with a dual profile; however, careful translational evaluation should address dosing windows that balance tumor sensitization with organ protection.

#### 3.5.8 Withaferin A

##### 3.5.8.1 Tumor antiangiogenic/Anticancer actions

Withaferin A, a steroidal lactone derived from *Withania somnifera* (ashwagandha), has demonstrated potent antiangiogenic effects. It can inhibit VEGF-induced angiogenesis-related endothelial activities, including migration, proliferation, and tube formation, and it has been reported to downregulate VEGF expression and additional proangiogenic mediators (Saha et al., 2013; Lee and Choi, 2016; Atteeq, 2022). Previous documented works reported that withaferin A enhances the potency of anticancer therapies (Sari et al., 2020; Yang et al., 2011). Szydlak, (2024) reported that withaferin A potentiates the effect of gemcitabine in reducing the growth of pancreatic cancer (Szydlak, 2024). Withaferin A potentiates the efficiency of cisplatin in managing ovarian cancer growth (Kakar et al., 2012).

##### 3.5.8.2 Organ cytoprotection during chemotherapy-related injury

**FIGURE 11:**
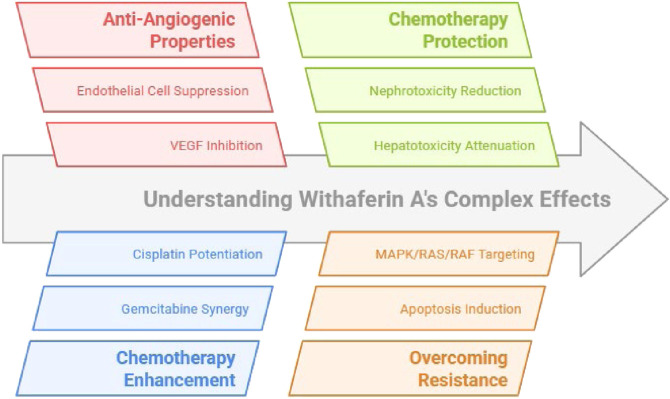
Withaferin dual role in cancer treatment and protection of vital organs.

Withaferin A has also been studied for protective effects against treatment-related cytotoxic injury in non-cancerous tissues. It has been reported to attenuate cisplatin-induced nephrotoxicity. In addition, withaferin A exhibits a protective effect against chemotherapy that causes cytotoxicity. Withaferin A attenuates cisplatin-induced nephrotoxicity in rats (Sharma et al., 2017). Mansour and Hafer, (2012) disclosed that withaferin A attenuates radiation-induced hepatotoxicity (Mansour and Hafez, 2012) (Figure 11).

##### 3.5.8.3 Combination with chemotherapy

Collectively, these findings position withaferin A as a candidate adjunct with both tumor-directed antiangiogenic activity and evidence of tissue protection in injury models. Future work should clarify optimal schedules and define safety boundaries, particularly where ROS-related mechanisms contribute to anticancer activity.

### 3.6 Potential applications in cancer treatment

The incorporation of phytochemicals into cancer treatment strategies offers several promising therapeutic advantages. Compared with conventional chemotherapeutic agents, phytochemicals generally exhibit lower toxicity, which makes them particularly attractive for long-term administration or use as adjunctive therapies. Their favorable safety profiles may help reduce the adverse effects commonly associated with standard cancer treatments, thereby improving patient tolerance and overall quality of life. In addition, the development of drug resistance remains a major limitation of many anti-angiogenic therapies. Due to their ability to modulate multiple molecular targets and signaling pathways, phytochemicals may help overcome or delay the emergence of resistance mechanisms in tumors, enhancing treatment durability and efficacy.

Furthermore, phytochemicals have demonstrated the potential to exert synergistic effects when combined with conventional anticancer therapies, including chemotherapy and targeted agents. Such combinations may amplify anticancer activity while simultaneously mitigating treatment-related toxicity. For example, studies have shown that the co-administration of curcumin with chemotherapeutic drugs can enhance therapeutic efficacy while reducing adverse side effects. Importantly, this synergism may also enable dose reduction of cytotoxic agents without compromising therapeutic outcomes, thereby decreasing systemic toxicity and improving patient quality of life.

### 3.7 Challenges and considerations

Despite the considerable promise of plant-derived phytochemicals as anti-angiogenic and cytoprotective agents in cancer therapy, several challenges must be addressed before their widespread clinical application can be realized. One of the primary limitations is their poor bioavailability, as many phytochemicals, including curcumin and resveratrol, exhibit low aqueous solubility, limited intestinal absorption, rapid metabolism, and fast systemic elimination. These pharmacokinetic constraints significantly reduce their therapeutic efficacy *in vivo*. To overcome these limitations, current research efforts are focused on the development of advanced drug delivery strategies, such as nanoparticle-based systems, liposomal formulations, and chemical modifications, which aim to enhance stability, bioavailability, and targeted tissue delivery.

Another critical challenge relates to standardization and dosing consistency. The biological activity of phytochemicals can vary substantially depending on their botanical source, extraction method, formulation, and administered dose. This variability complicates the reproducibility of experimental findings and hinders the translation of preclinical results into consistent clinical outcomes. The establishment of standardized extraction protocols, well-defined formulations, and evidence-based dosing regimens is therefore essential to ensure reliability and comparability across studies and to support their integration into clinical practice.

Furthermore, there remains a significant need for robust clinical evidence to validate the efficacy and safety of phytochemicals in human cancer therapy. While numerous *in vitro* and *in vivo* studies have demonstrated their anti-angiogenic and cytoprotective potential, well-designed clinical trials are required to confirm these effects in patients. The transition from laboratory research to clinical application necessitates careful evaluation of pharmacokinetics, potential interactions with conventional anticancer therapies, inter-individual variability, and long-term safety profiles. Addressing these considerations will be crucial for determining the clinical utility of phytochemicals and for facilitating their successful incorporation into evidence-based cancer treatment strategies.

## 4 Discussion

The therapeutic targeting of angiogenesis in oncology has evolved from a broad-spectrum anti-vascular strategy to a more nuanced paradigm that recognizes the contextual duality of blood vessel regulation (Liu et al., 2023). This review underscores a critical dichotomy: while pathological angiogenesis is a cornerstone of tumor progression and metastasis, physiological angiogenesis remains essential for the repair and protection of healthy tissues, especially those under the assault of cytotoxic chemotherapy. The emerging role of phytochemicals as modulators of both processes offers a sophisticated therapeutic approach, simultaneously dismantling the tumor’s lifelines while fortifying the patient’s vital organs (Sa et al., 2023).

Our analysis confirms that phytochemicals like curcumin, resveratrol, EGCG, and others exert potent anti-angiogenic effects primarily through the suppression of master regulators such as VEGF, HIF-1α, and NF-κB (Zhang et al., 2021; Huang et al., 2017; Delmas et al., 2011; Fu et al., 2021; Yamakawa et al., 2004; Demeule et al., 2000). This is not merely a laboratory observation; it translates to a tangible disruption of the tumor’s complex signaling network. For instance, the ability of EGCG to inhibit MMP-2/9 activity or sulforaphane to modulate HDAC highlights how these compounds attack angiogenesis at multiple, convergent nodes. This polypharmacology is a key advantage over single-target synthetic anti-angiogenics, as it inherently raises the barrier for the development of tumor resistance, a frequent and debilitating limitation of drugs like bevacizumab.

The more profound insight, however, lies in their context-dependent proangiogenic capacity. In the milieu of healthy tissues stressed by chemotherapy, where endothelial dysfunction, oxidative damage, and inflammation prevail, these same compounds appear to switch roles. They promote vascular health and repair, likely through the upregulation of endogenous antioxidant pathways (e.g., Nrf2/HO-1), reduction of inflammatory cytokines, and mitigation of apoptosis in non-cancerous endothelial cells. This is not a contradiction but a reflection of biological intelligence (Banerjee et al., 2016). For example, the observation that EGCG normalizes chaotic tumor vasculature yet supports angiogenesis in ischemic cardiac or skeletal muscle is a testament to its tissue-selective modulatory capacity (Ibrahim et al., 2019). This dual function addresses one of the most significant challenges in medical oncology: how to protect the patient without protecting the tumor.

From a clinical translation perspective, this duality positions phytochemicals uniquely as chemosensitizers and chemo-protectors. They can enhance the efficacy of first-line agents, such as cisplatin or doxorubicin, by overcoming hypoxia-driven resistance and improving drug delivery through vascular normalization, while concurrently shielding the heart, liver, and kidneys from dose-limiting toxicities. This could potentially enable more aggressive or prolonged treatment regimens, thereby improving outcomes. However, the path from this compelling mechanistic rationale to clinical practice is fraught with real-world hurdles that our field has grappled with for decades (Zahedi et al., 2023).

The foremost challenge remains bioavailability. The promising *in vitro* and animal data for compounds like curcumin and resveratrol have often stumbled at the human pharmacokinetic gate. However, translation to humans is constrained by limited oral bioavailability and rapid metabolism for several compounds, necessitating optimized formulations (e.g., lipid carriers, nanoparticles, phospholipid complexes) to achieve therapeutically relevant exposures. Recent work by researchers like Sardou et al. (2023) on curcumin-loaded nanostructured lipid carriers for colorectal cancer models exemplifies the innovative formulations required to bridge this gap (Sardou et al., 2023).

Furthermore, standardization and dosing are not merely academic concerns. The biological activity of a plant extract can vary dramatically based on cultivation, extraction method, and storage. Moving forward, clinical trials must employ standardized, well-characterized botanical drug substances, as defined by regulatory bodies like the FDA. The dosing question is equally complex; more is not always better, and the biphasic, hormetic effects common to many phytochemicals necessitate carefully calibrated “therapeutic windows” that are likely disease- and patient-specific.

Finally, while preclinical evidence is robust, the clinical evidence remains nascent. Most human studies to date are small-scale phase I/II trials. There is a pressing need for large, rigorous, randomized controlled trials (RCTs) that evaluate these agents not as alternatives, but as sophisticated adjuncts to standard-of-care. Recent trials, such as those investigating the combination of curcumin with FOLFOX in metastatic colorectal cancer (NCT05507636), are beginning to provide this crucial human data. The field must also embrace biomarker-driven studies to identify which patients, based on their tumor vasculature profile or genetic makeup, are most likely to benefit from this integrative approach.

### 4.1 Future directions and computational discovery

Dual angiogenesis modulation is a testable framework rather than a descriptive label. Future studies should prioritize methods that can identify compounds with tumor-selective antiangiogenic signatures while preserving endothelial resilience in normal tissues. Computational approaches, such as network pharmacology, target-pathway enrichment, and docking-based prioritization, can integrate phytochemical target profiles with angiogenesis and organ-injury pathways to nominate candidates for validation. This should be coupled with multi-omics or single-cell analyses in tumor versus organ-injury models to confirm context-dependent signaling effects and to define biomarkers that distinguish beneficial organ protection from undesirable tumor support.

## 5 Conclusion

This review consolidates evidence supporting a dual-modulation concept in which selected phytochemicals may suppress pathological tumor angiogenesis while supporting cytoprotection, endothelial resilience, and microvascular recovery in chemotherapy-injured vital organs. The clinical importance of this framework lies in its alignment with a central oncologic goal: improving tumor control while reducing dose-limiting toxicity that compromises treatment intensity, duration, and patient quality of life.

To translate this concept into practice, future work should prioritize three steps: (i) development of reliable, bioavailable formulations and delivery strategies that achieve reproducible systemic exposure; (ii) standardization of preparations and dosing schedules, with explicit definition of therapeutic windows; and (iii) biomarker-guided clinical trial designs that evaluate tumor outcomes and organ protection endpoints simultaneously to ensure that normal-tissue protection does not come at the expense of antitumor efficacy. With these elements in place, phytochemicals can be evaluated not as “alternative” therapies, but as scientifically grounded adjuncts that may expand the effectiveness and tolerability of modern cancer treatment.
